# C2-O-sLe^X^ Glycoproteins Are E-Selectin Ligands that Regulate Invasion of Human Colon and Hepatic Carcinoma Cells

**DOI:** 10.1371/journal.pone.0016281

**Published:** 2011-01-19

**Authors:** Catherine A. St. Hill, Dahabo Baharo-Hassan, Mariya Farooqui

**Affiliations:** Department of Veterinary Clinical Sciences, University of Minnesota, St. Paul, Minnesota, United States of America; University of Bergen, Norway

## Abstract

Similar to mechanisms of recruitment of activated leukocytes to inflamed tissues, selectins mediate adhesion and extravasation of circulating cancer cells. Our objective was to determine whether sialyl Lewis X modified core 2 O-glycans (C2-O-sLe^X^) present on colon and hepatic carcinoma cells promote their adhesion and invasion. We examined membrane expression of C2-O-sLe^X^, selectin binding, invasion of human colon and hepatic carcinoma cell lines, and mRNA levels of alpha-2,3 fucosyltransferase (FucT-III) and core 2 beta-1,6 N-acetylglucosaminyltransferase (C2GnT1) genes, necessary for C2-O-sLe^X^ synthesis, by quantitative reverse-transcriptase (RT) PCR. Synthesis of core 2 branched O-glycans decorated by sLe^X^ is dependent on C2GnT1 function and thus we determined enzyme activity of C2GnT1. The cell lines that expressed C2GnT1 and FucT-III mRNA by quantitative RT-PCR were highly positive for C2-O-sLe^X^ by flow cytometry, and colon carcinoma cells possessed highly active C2GnT1 enzyme. Cells bound avidly to E-selection but not to P- and L-selectin. Gene knock-down of C2GnT1 in colon and hepatic carcinoma cells using short hairpin RNAs (shRNA) resulted in a 40–90% decrease in C2-O-sLe^X^ and a 30–50% decrease in E-selectin binding compared to control cells. Invasion of hepatic and colon carcinoma cells containing C2GnT1 shRNA was significantly reduced compared to control cells in Matrigel assays and C2GnT1 activity was down-regulated in the latter cells. The sLe^X^ epitope was predominantly distributed on core 2 O-glycans on colon and hepatic carcinoma cells. Our findings indicate that C2GnT1 gene expression and the resulting C2-O-sLe^X^ carbohydrates produced mediate the adhesive and invasive behaviors of human carcinomas which may influence their metastatic potential.

## Introduction

Recognition and binding of selectins to sialyl Lewis X (sLe^X^) and related oligosaccharides are crucial interactions that regulate leukocyte adhesion to blood vessels and extravasation into tissues in an inflammatory response [1]. Similar mechanisms are used by circulating tumor cells during metastasis to enter target organs [2], [3]. Selectin adhesion molecules: L-selectin constitutively expressed on most leukocytes [4], P-selectin expressed in activated platelets and endothelial cells [5], [6], and E-selectin induced on cytokine stimulated endothelial cells [7], mediate these processes. High affinity binding of selectins to sLe^X^ on human leukocytes is greatly enhanced when sLe^X^ is terminally displayed on core 2 based O-linked glycans (C2-O-sLe^X^) [8], [9], [10]. On leukocytes, C2-O-sLe^X^ decorates the mucin P-selectin glycoprotein ligand-1 (PSGL1, CD162) and is particularly important for high-strength binding interactions with P-selectin [11], [12], [13], [14].

Synthesis of carbohydrates with sialyl Lewis structures increases upon neoplastic transformation and are useful markers for cancers [15]. Several carcinomas including colon, gastric, lung, pancreatic, prostate, and urinary bladder highly express the selectin ligand sLe^X^ and expression is significantly correlated with advanced disease and a poor prognosis [16], [17], [18], [19], [20], [21]. Up-regulation of vascular E-selectin in cancers with high expression of sialyl Lewis structures is thought to be a risk factor for hematogenous metastasis [22]. In colon and hepatocellular carcinomas, a high content of sLeX antigens is associated with increased metastatic potential [17], [23] but the molecular mechanisms involving sLeX that regulate metastasis are not well understood. It is important to understand the role of glycans in cancer progression because the altered N- or O-glycosylation status of tumors may predict their metastatic potential and promote invasion and metastasis [24]. We have previously demonstrated that a colon carcinoma cell line expressing C2-O-sLe^X^ carbohydrates binds to E-selectin [25], and that C2-O-sLe^X^ is a tumor-associated antigen in colon cancer tissues [26]. In this report, we investigated the impact of disrupting C2-O-sLe^X^ synthesis on the distribution of sLe^X^ on core 2 O-glycans versus N-glycans on colon and hepatic carcinoma cell lines, their E-selectin binding capacity, and the influence on their invasive properties as initial steps to elucidate the function of these carbohydrates in metastasis.

**Figure 1:**
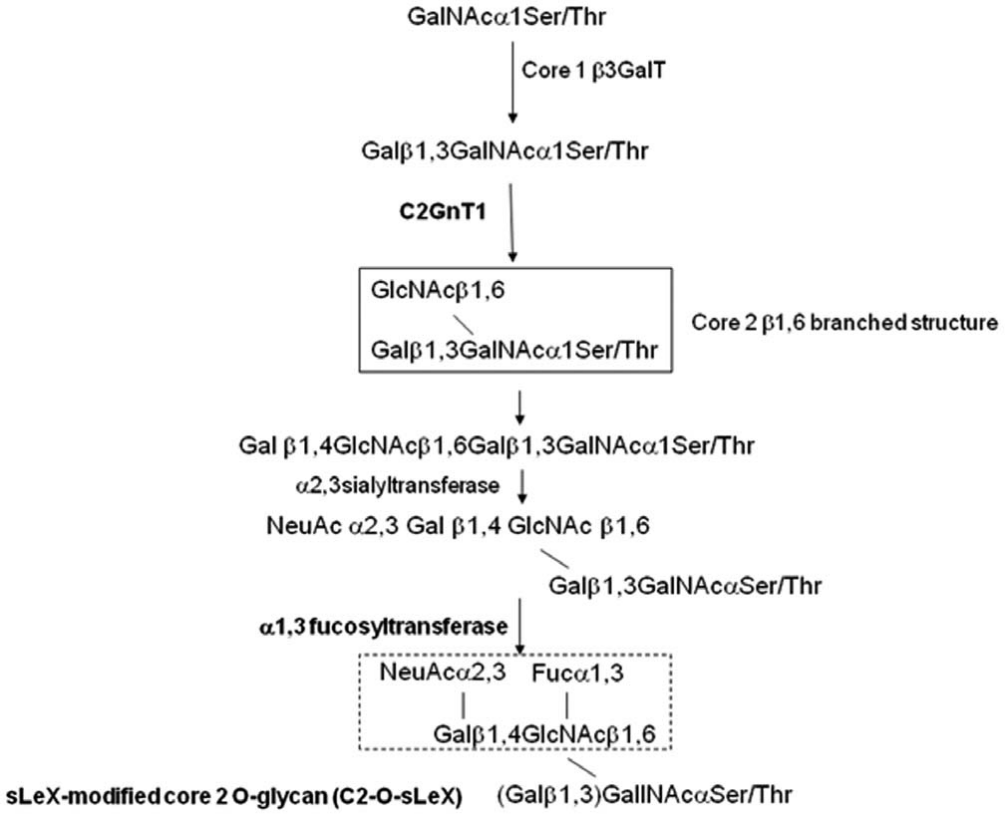
Pathway of C2-O-sLe^X^ biosynthesis. A simplified version of C2-O-sLe^X^ biosynthesis is shown. Core 1 O-glycans are synthesized by addition of β1,3 galactose to N-acetylgalactosamine. The core 2 β1,6-*N*-acetylglucosaminyltransferase (C2GnT1) enzyme catalyzes the addition of a β1,6 branch to a core 1 O-glycan to form a core 2 β1,6 branched O-glycan. The core 2 O-glycan structure is further modified by a series of enzymatic reactions (omitted for clarity) including modification by α2,3 sialyltransferase and α1,3 fucosyltransferase (FucT-III) to form a sLe^X^ terminus (dotted box). These modifications result in the synthesis of the sLe^X^-modified core 2 β1,6 O-glycan (C2-O-sLe^X^) structure. GalNAc, N-acetylgalactosamine; Gal, galactose; GlcNAc, N-acetylglucosamine; NeuAc, sialic acid; Fuc, fucose.

C2-O-sLe^X^ biosynthesis is complex and occurs by a series of enzymatic steps involving activities of glycosyltransferases (Fig. 1). Briefly, N-acetylgalactosamine is added to serine or threonine residues at the protein backbone, followed by galactose in a β1,3 linkage, creating the core 1 extension. The enzyme core 2 β1,6-N-acetylglucosaminyltransferase (C2GnT1) initiates the core 2 extension by adding N-acetylglucosamine to N-acetylgalactosamine in a β1,6 linkage and is the key branching enzyme in core 2 O-glycan biosynthesis. Core 2 branches are elongated by several glycosyltransferases and terminated by the addition of sialic acid in an α2,3 linkage to galactose. Finally, α(1,3/1,4) fucosyltransferase (FucT-III) catalyzes the addition of fucose to the chain in an α1,3 linkage, resulting in the formation of C2-O-sLe^X^ [27]. Functional activity of both C2GnT1 and FucT-III enzymes is necessary for C2-O-sLe^X^ synthesis [28], [29], [30].

We targeted the C2GnT1 gene to disrupt C2-O-sLe^X^ biosynthesis in carcinoma cells. We chose this gene because high C2GnT1 expression in carcinomas has previously been correlated with vessel invasion, depth of tumor invasion, and metastasis [31], [32], [33]. Using the CHO-131 monoclonal antibody (mAb) which specifically detects C2-O-sLe^X^ [28], we directly examined the ability of tumor cells transduced with C2GnT1 shRNA and expressing low levels C2-O-sLe^X^ to bind to selectins. We have previously shown that C2-O-sLe^X^ is a tumor-associated ligand that is abundant on the invasive front of human colon carcinoma tissues, and mediates E-selectin binding when expressed on a colon carcinoma cell line [25], [26]. In this report, we directly demonstrated for the first time that expression and activity of the C2GnT1 gene responsible for C2-O-sLe^X^ synthesis in colon and hepatic carcinoma cell lines regulated invasion of tumor cells, a key property that facilitates metastasis. We found that the sLe^X^ epitope was predominantly distributed on O-glycans compared to N-glycans and was an important E-selectin ligand in these cell lines.

## Results

### Human carcinoma cell lines highly express genes involved in C2-O-sLe^X^ synthesis and bind to E-selectin

**Figure 2:**
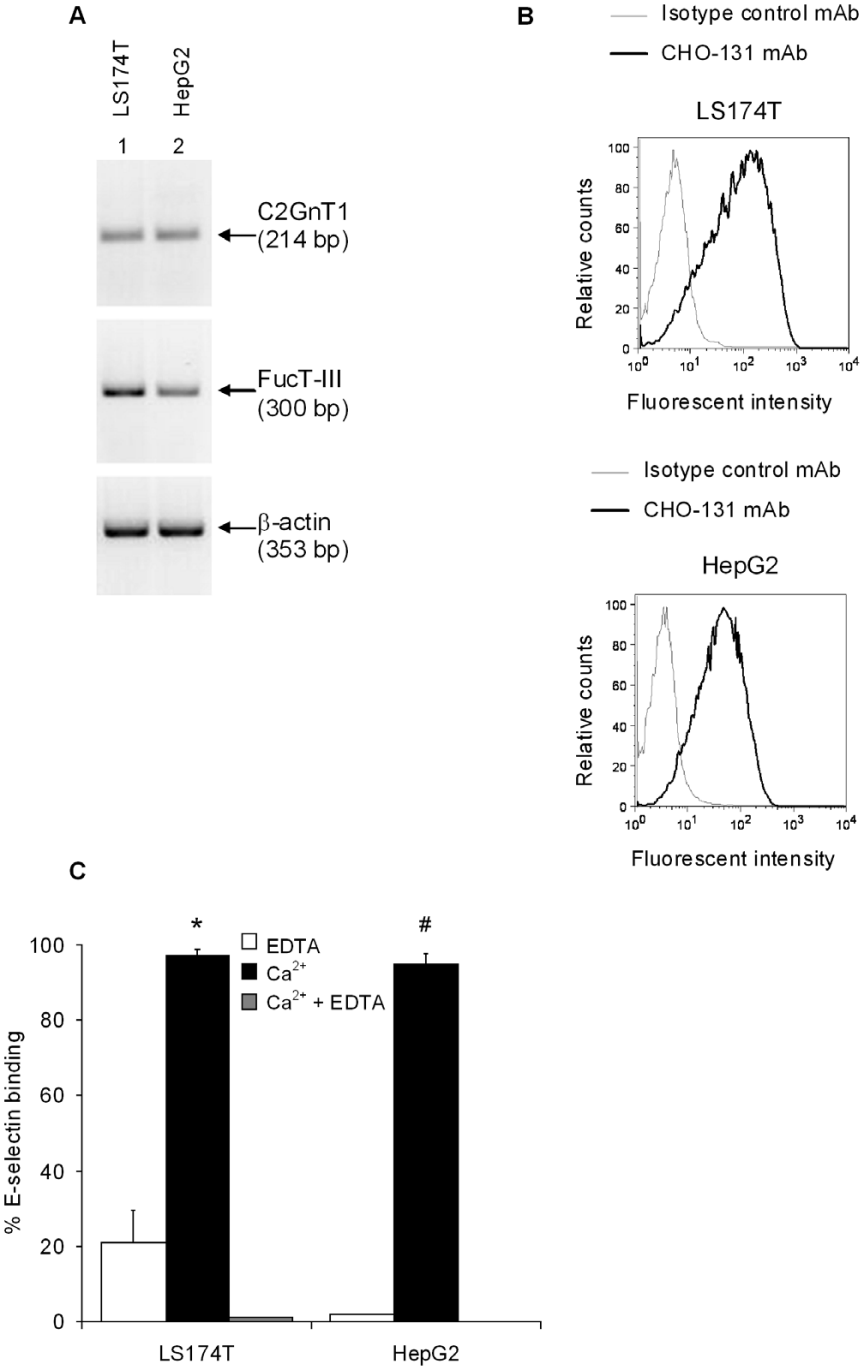
C2GnT1 and FucT-III genes are endogenously expressed in human carcinoma cell lines. (A) Endogenous C2GnT1 and FucT-III mRNA transcripts in LS174T colorectal adenocarcinoma (lane 1) and HepG2 hepatic carcinoma (lane 2) cell lines were detected by RT-PCR. (B) Flow cytometric analysis of positive staining of LS174T and HepG2 cells labeled with CHO-131 mAb (anti-C2-O-sLe^X^). A representative example of 3 experiments is shown for (A-B). (C) LS174T (*p = 0.008) and HepG2 cells (#p = 0.001) substantially bind to E-selectin in the presence of calcium ions (Ca^2+^). Binding is impeded in the presence of EDTA, a calcium ion chelator. The average of two experiments is shown.

We examined mRNA expression of C2GnT1 and FucT-III genes in colon (LS174T) and hepatic (HepG2) cell lines by RT-PCR. Both cell lines expressed C2GnT1 and FucT-III mRNA necessary for C2-O-sLe^X^ synthesis (Fig. 2A). To investigate whether mRNA expression of the C2GnT1 and FucT-III genes resulted in cell surface expression of C2-O-sLe^X^, we tested the carcinoma cell lines for reactivity with CHO-131 mAb by flow cytometry. Approximately 75% of un-manipulated LS174T colorectal adenocarcinoma cells (MFI, 5866) and 55% of HepG2 hepatic carcinoma cells (MFI, 1659) reacted positively with CHO-131 mAb but did not react with an isotype control mAb (Fig. 2B).

The binding of the sLe^X^ epitope on colon carcinoma cells to E-selectin has been associated with a greatly increased metastatic potential and a poor prognosis [17]. We therefore examined the participation of C2-O- sLe^X^ in E-selectin binding of LS174T and HepG2 cells by detecting the ability of these carcinoma cell lines expressing C2-O-sLe^X^ to bind to a mouse E-selectin Fc chimera by flow cytometry. Selectin binding interactions are calcium dependent and are dissociated in the presence of EDTA, a chelator of calcium ions. LS174T and HepG2 cells with high endogenous C2-O-sLe^X^ expression bound avidly to E-selection (98% and 95% cellular binding for LS174T and HepG2 cells respectively compared to binding in the presence of 10 mM EDTA) (Fig. 2C).

### Knock-down of the C2GnT1 gene results in decreased E-selectin binding

Currently, a specific inhibitor of C2GnT1 expression and activity is not available. To directly evaluate the influence of C2GnT1 activity on the generation of C2-O-sLe^X^ epitopes on tumor cells and the binding to E-selectin, we instead used short hairpin RNA (shRNA) gene silencing techniques to target the C2GnT1 gene in HepG2 and LS174T cells. The four lentiviral pGIPZ-C2GnT1-shRNA vectors, the PLKO.1-C2GnT-shRNA vector, the scrambled pGIPZ-shRNA vector, and the empty PLKO.1 control vector were tested for silencing of the C2GnT1 gene in LS174T colon carcinoma and HepG2 hepatic carcinoma cells. All shRNA sequences achieved consistent gene knockdown in the cell lines tested indicating that our observed effects were specifically due to loss of the C2GnT1 gene. The clones that were most efficient at gene knockdown were selected for subsequent experiments. Thus, LS174T cells were stably transduced with one of the lentiviral pGIPZ-C2GnT1-shRNA vectors or with a scrambled pGIPZ-shRNA vector. HepG2 cells were transiently transfected with the lentiviral PLKO.1-C2GnT-shRNA vector or with an empty PLKO.1 control vector. The C2GnT1 shRNA sequences were homologous to only the human C2GnT1 gene when compared to the human genome using NCBI Nucleotide BLAST. The scrambled shRNA vectors were not complementary to any gene in the human genome.

**Figure 3:**
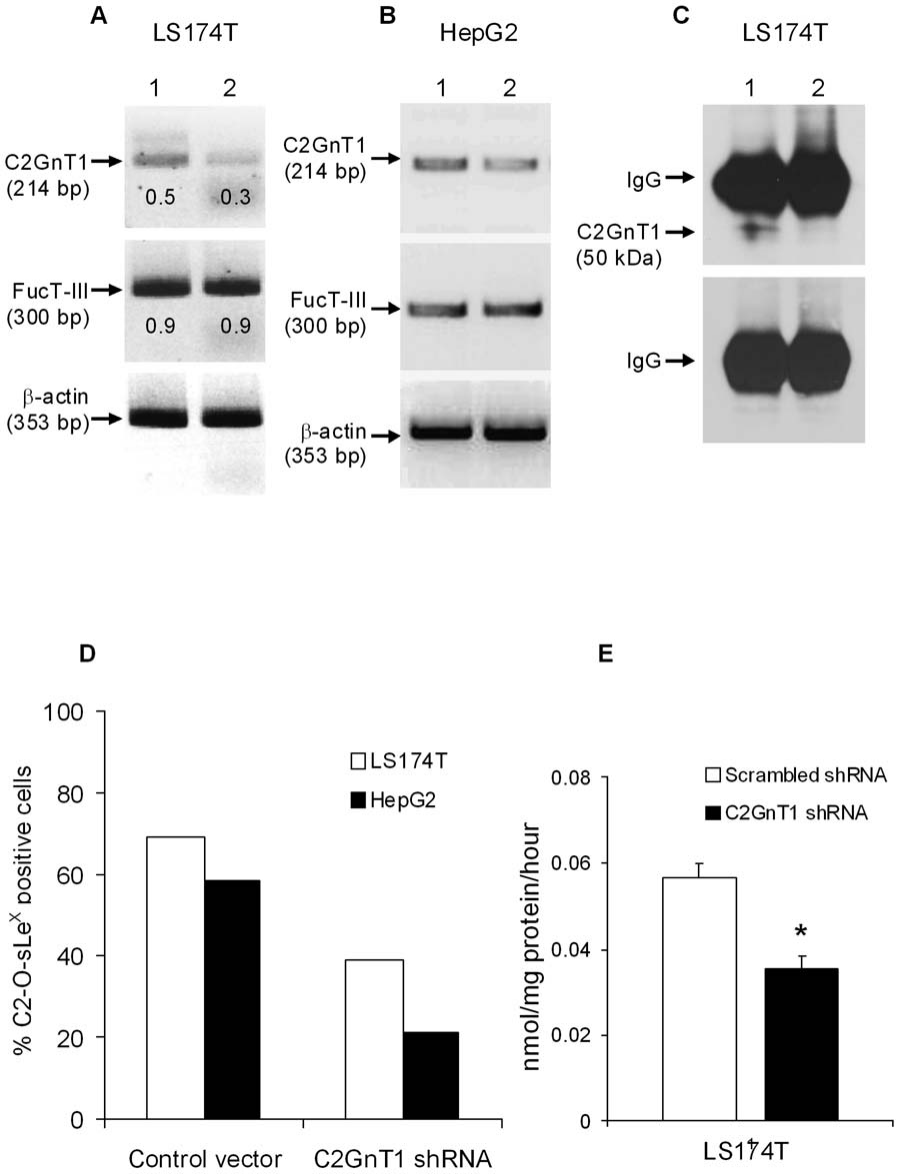
C2GnT1 gene knockdown decreases C2-O-sLe^X^ glycoproteins and C2GnT1 activity. (A) C2GnT1 suppression by shRNA resulted in decreased mRNA levels of C2GnT1 but not FucT-III in LS174T cells, (scrambled shRNA vector, lane 1, and C2GnT1 shRNA vector, lane 2). (B) Similar effects on C2GnT1 mRNA but not FucT-III mRNA were observed in HepG2 cells after transient transfection with C2GnT1 shRNA (empty control vector, lane 1, and targeting shRNA vector, lane 2). Densitometry values are normalized to β-actin. (C) The C2GnT1 glycoprotein (50 kDa) was present in scrambled shRNA transduced LS174T cells (lane 1) but absent in C2GnT1 shRNA transduced cells (lane 2) as detected by immunoprecipitation and Western blotting using a polyclonal anti-C2GnT1 antibody. (D) The procedures were repeated using the C2GnT1 antibody for immunoprecipitation and a negative control IgG antibody for Western blotting. Note the absence of a detected C2GnT1 protein 50 kDa band. (D) We observed a 30–40% decrease in reactivity with CHO-131 mAb by flow cytometry for LS174T and HepG2 cells that contained C2GnT1 shRNA. Representative data from multiple experiments are shown. (E) C2GnT1 enzyme activity, measured as nmols of N-acetylglucosamine (GlcNAc) transferred per mg protein per hour in a glycosyltransferase assay, was also significantly decreased in C2GnT1 shRNA transduced LS174T cells, *p = 0.02. Mean values ± standard deviations of two independent experiments are presented.

As shown in Fig. 3A, mRNA levels of the C2GnT1 gene were considerably lower in LS174T cells transduced with C2GnT1 shRNA compared to cells transduced with the scrambled shRNA vector by densitometry. Similarly, mRNA levels of C2GnT1 were lower in HepG2 cells transfected with C2GnT1 shRNA compared to cells transfected with the control vector (Fig. 3B). In contrast, in both cell lines, FucT-III mRNA levels were not affected by silencing of the C2GnT1 gene, indicating that the shRNA clones did not have off-target effects. The C2GnT1 protein was detected in LS174T cells transduced with the scrambled shRNA vector but not in cells transduced with C2GnT1 shRNA after immunoprecipitation and Western blotting with an anti-C2GnT1 antibody (Fig. 3C and D). The percentage of cells that was reactive with CHO-131 mAb, indicating expression of cell surface C2-O-sLe^X^ before and after C2GnT1 gene knock-down, was assessed by flow cytometry. In multiple experiments using LS174T and HepG2 cells, we consistently achieved approximately 30–40% reduction of C2-O-sLe^X^ expression after C2GnT1 gene knock-down and a representative experiment is shown in Fig. 3D. We verified silencing of the C2GnT1 gene by examining C2GnT1 enzyme activity in LS174T cells and observed a significant ∼40% decrease in enzyme activity of LS174T cells transduced with C2GnT1 shRNA compared to those cells transduced with scrambled shRNA (p = 0.02, Fig. 3E). For all groups of cells, viability was assessed by trypan blue exclusion and was greater than 95%.

**Figure 4:**
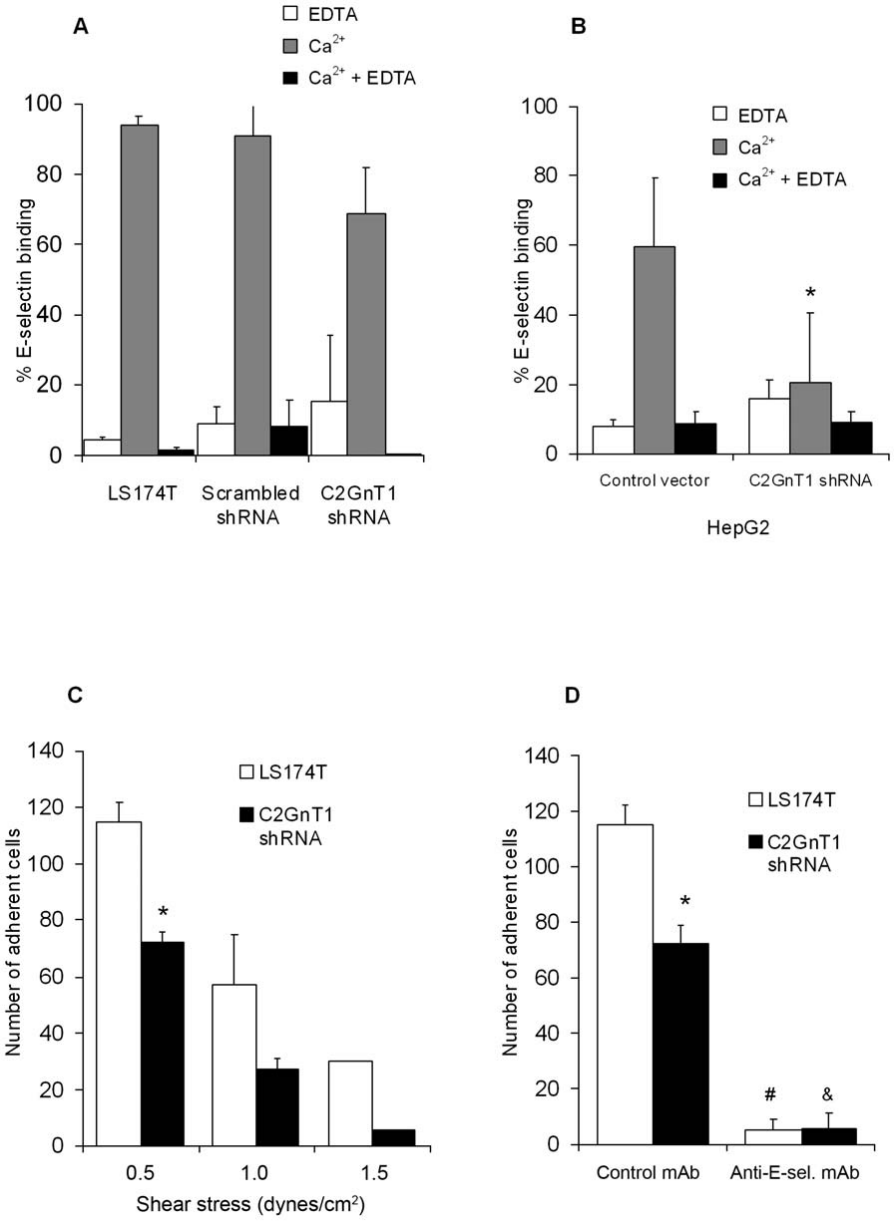
C2GnT1 gene knock-down results in decreased binding of cells to E-selectin. (A) Significant differences in E-selectin binding were not observed among LS174T un-manipulated cells, cells transduced with scrambled shRNA, or with C2GnT1 shRNA by flow cytometry. (B) E-selectin binding was significantly decreased for HepG2 cells transfected with C2GnT1 shRNA compared to cells transfected with the control vector, *p = 0.02. (C) Shear flow assays were performed at an E-selectin/Fc chimera concentration of 1 µg/ml and shear stresses ranging from 0.5 – 1.5 dynes/cm^2^ in the presence of 10 µg/ml of an IgG isotype control mAb. At a shear stress of 0.5 dynes/cm^2^, significantly fewer C2GnT1 shRNA transduced cells accumulated on E-selectin than un-manipulated LS174T cells, *p = 0.04. (D) In the same run of experiments, at a shear stress of 0.5 dynes/cm^2^, significantly fewer un-manipulated LS174T cells treated with a 10 µg/ml of a blocking anti-E-selectin mAb accumulated on E-selectin than those cells treated with an isotype control mAb, ^#^p = 0.004. Significantly fewer LS174T cells transduced with C2GnT1 shRNA and treated with a blocking anti-E-selectin mAb accumulated on E-selectin than those cells treated with an isotype control mAb, ^&^p = 0.01. The same assay as shown in (C) at 0.5 dynes/cm^2^ is included for comparison. For both groups of cells treated with an isotype control mAb, significantly fewer un-manipulated LS174T cells accumulated on E-selectin than LS174T cells transduced with C2GnT1 shRNA, *p = 0.04. Each continuous shear flow assay was performed in duplicate for each shear stress and the bars represent the mean ± standard deviations. Representative data from three independent experiments are shown.

For LS174T and HepG2 cells in which the C2GnT1 gene was silenced and for control cells, reactivity with a mouse E-selectin/Fc chimera was assessed by flow cytometry. We did not observe a significant decrease in E-selectin binding of LS174T cells containing C2GnT1 shRNA compared to un-manipulated cells and cells transduced with scrambled shRNA (Fig. 4A). However, we observed a significant 40% decrease in E-selectin binding for C2GnT1 shRNA-transfected HepG2 cells compared to those cells transfected with the control vector, (p = 0.02, Fig. 4B).

To further assess the influence of C2GnT1 expression on E-selectin binding of LS174T cells, we performed more stringent hydrodynamic shear flow assays that closely simulate forces occurring in the microvasculature. The adhesive behavior of cells was examined at a range of shear stresses between 0.5 and 1.5 dynes/cm^2^ and chimera concentrations between 0.1 and 5 µg/ml in the presence of 10 µg/ml of an anti-E-selectin function-blocking mAb or an isotype-matched control mAb. We observed optimal E-selectin binding of isotype control mAb treated un-manipulated LS174T cells and LS174T cells transduced with C2GnT1 shRNA at a concentration of 1 µg/ml of the E-selectin/Fc chimera and a shear stress of 0.5 dynes/cm^2^ (Fig. 4C). Under these conditions, accumulation of LS174T cells transduced with C2GnT1 shRNA was reduced by 36% compared to accumulation of un-manipulated LS174T cells, p = 0.04. For both groups of cells, fewer cells accumulated on the E-selectin chimera as the shear stress was increased to 1.5 dynes/cm^2^. In order to assess the specificity of the interaction of cells with E-selectin, both groups of cells were exposed to an anti-E-selectin function-blocking mAb (10 µg/ml) or an isotype-matched control mAb at the same concentration. Accumulated cells at a shear stress of 0.5 dynes/cm^2^ are shown in Fig. 4D and binding of both groups of cells exposed to the isotype control mAb is shown for comparison. The presence of an E-selectin blocking mAb greatly diminished the numbers of un-manipulated LS1704T (p = 0.004) and C2GnT1 transduced cells bound to E-selectin (p = 0.0001). The addition of 20 mM EDTA to perfusates prevented the binding of cells to E-selectin (data not shown).

### LS174T and HepG2 cells predominantly express sLe^X^ on core 2 O-glycans

**Figure 5:**
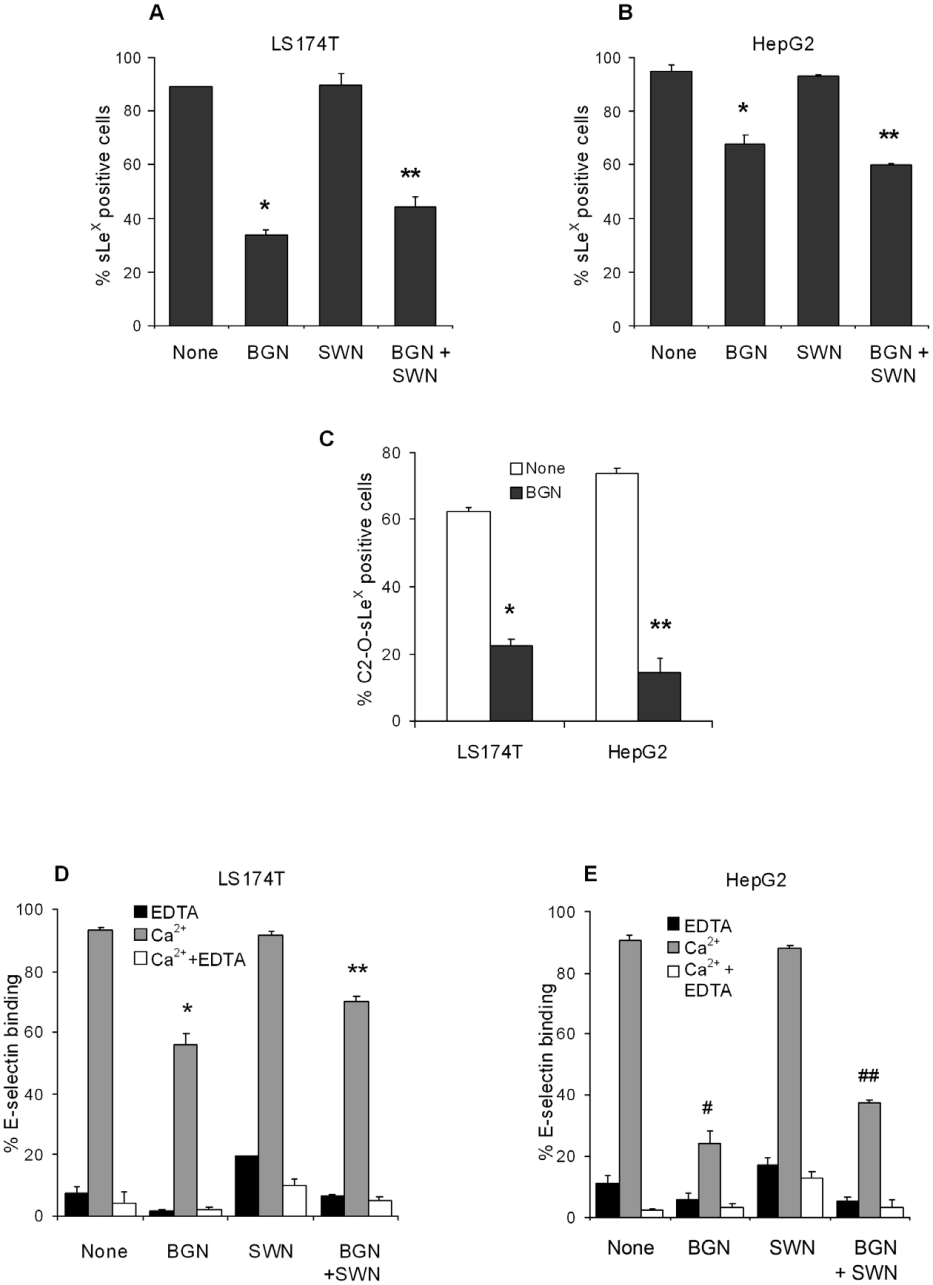
LS174T and HepG2 cells predominantly express sLe^X^ on core 2 O-glycans. (A) The percentage of sLe^X^ positive LS174T cells was significantly decreased after treatment with BGN (an O-glycosylation inhibitor) *p = 0.0004, or with BGN and SWN (an N-glycosylation inhibitor, **p = 0.002) compared to untreated cells (None). (B) For HepG2 cells, a reduction in the percentage of sLe^X^ positive cells was observed after treatment with BGN *p < 0.0001, or with BGN and SWN **p < 0.0001, compared to untreated cells. (C) The cells used in (A) and (B) before and after treatment with BGN were also labeled with CHO-131 mAb to specifically detect C2-O-sLe^X^. Note the significant reduction in the percentage of C2-O-sLe^X^ positive LS174T and HepG2 cells after treatment with BGN compared to untreated cells, * and **p < 0.0001. (D) A significant decrease in E-selectin binding was observed for LS174T cells after treatment with BGN *p = 0.003, and after BGN and SWN **p = 0.0005, compared to untreated cells. (E) Similar results were observed for HepG2 cells after BGN, ^#^p = 0.0005, and after BGN and SWN, ^##^p<0.0001, compared to untreated cells.

In order to determine the nature of the carbohydrate structure to which the sLe^X^ epitope is attached in un-manipulated LS174T and HepG2 cells, we treated cells with agents to inhibit O-glycosylation (BGN) and N-glycosylation (SWN) because these glycans are the main carriers of sLe^X^. Treated and untreated cells were labeled with CSLEX1 mAb that detects sLe^X^ on any structure to assess the percentage of sLe^X^ positive cells on O-glycans versus N-glycans by flow cytometry. For both LS174T and HepG2 cells in the presence of BGN but not SWN, a significant decrease in the percentage of cells reactive with CSLEX1 mAb was observed compared to untreated cells (Fig. 5A and B, p = 0.0004 for LS174T cells and p<0.0001 for HepG2 cells) indicating that sLe^X^ was mainly expressed on O-glycans in these cell types. Treatment of LS174T and HepG2 cells with both BGN and SWN inhibitors did not cause a further reduction in cells reactive with CSLEX1 mAb but the decrease was significant when compared to untreated cells, (p = 0.002 and p<0.0001 respectively), and was likely due to the effects of BGN.

LS174T and HepG2 cells, either untreated or treated with BGN, were subsequently labeled with CHO-131 mAb that specifically detects C2-O-sLe^X^ positive cells to determine the percentage of the subset of core 2 O-glycans that carried sLe^X^. We observed that reactivity with CHO-131 mAb was significantly reduced by 40% in BGN-treated LS174T and by 60% in HepG2 cells compared to untreated cells (Fig. 5C, p<0.0001). Binding to E-selectin was also evaluated in untreated cells and in cells treated with the inhibitors. As shown in Fig. 5D and E, BGN treatment significantly decreased E-selectin binding of LS174T cells by 38% (p = 0.003) and of HepG2 cells by 67% (p = 0.0005) compared to untreated cells. A combination of BGN and SWN treatment did not result in further reductions in E-selectin binding of LS174T and HepG2 cells but significant differences were observed when compared to untreated cells, p = 0.0005 and p<0.0001 respectively, indicating the effects of BGN treatment.

### C2-O-sLe^X^ mediates the invasion of carcinoma cells

**Figure 6:**
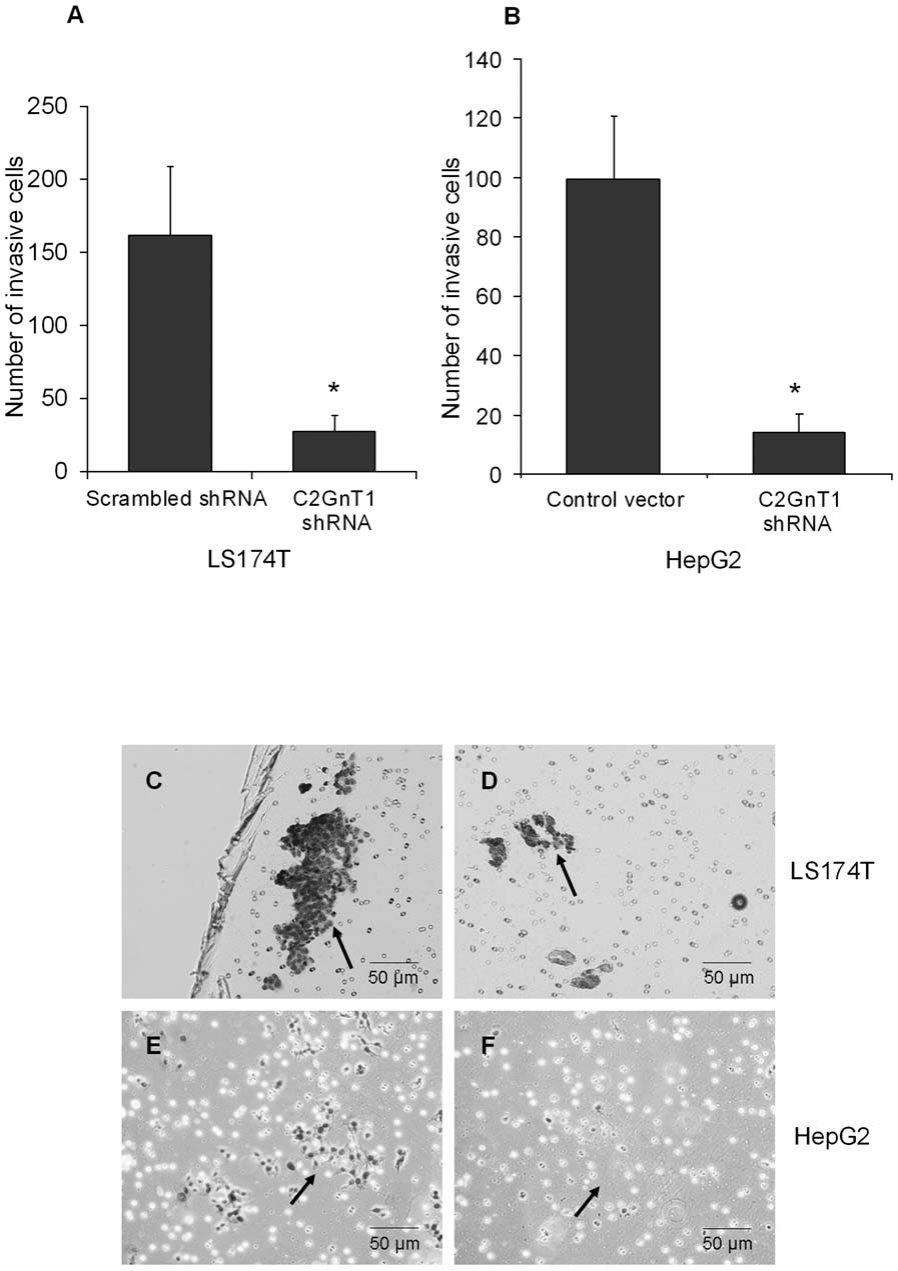
C2GnT1 mediates invasion. (A) LS174T cells transduced with C2GnT1 shRNA were significantly less invasive than cells transduced with scrambled shRNA, *p<0.0001, in Matrigel transwell invasion assays. (B) Similarly, decreased invasion was observed for HepG2 cells that were transfected with C2GnT1 shRNA compared to transfection with a control vector, *p = 0.0005. (C) Representative photomicrographs from two separate experiments of invasion of LS174T cells transduced with the scrambled shRNA vector compared to (D) cells transduced with C2GnT1 shRNA and (E) HepG2 cells transfected with the control vector compared to (F) HepG2 cells transfected with C2GnT1 shRNA. The invading cells were stained and counted in five separate fields of view at 100X magnification. Arrows indicate invasive cells.

We examined the influence of silencing of the C2GnT1 gene on the invasive properties of LS174T and HepG2 carcinoma cells. Using a Matrigel invasion assay that mimics active invasion of tumor cells across a basement membrane, we found that after 48 hours, the number of C2GnT1 shRNA-transduced LS174T cells that invaded across the Matrigel membrane was significantly decreased compared to LS174T cells transduced with scrambled shRNA (mean number of cells/field  = 27 versus 162, p<0.0001) (Fig. 6A, C, and D). Similarly, we observed a significant decrease in invasion of HepG2 cells transfected with C2GnT1 shRNA compared to cells transfected with a control vector, (mean number of cells/field  = 14 versus 99, p<0.0005) (Fig. 6B, E, and F). Our results indicated that C2GnT1 gene expression mediated the invasive properties of LS174T and HepG2 cells by an unknown mechanism.

## Discussion

In the metastatic process, circulating cancer cells in the bloodstream arrest and adhere to endothelium, extravasate and invade into organs distant from the primary tumor, survive, proliferate, and promote angiogenesis. These multistep events require complex adhesive interactions between ligands on cancer cells and cellular adhesion molecules and other substrates present in the tumor microenvironment [21], [34]. Many types of cancers over-express altered carbohydrates that profoundly impact tumor growth, adhesion, invasion, and metastasis. Alterations in glycosylation, including the production of sLe^X^ epitopes, are generated by glycosyltransferases, yet surprisingly the role of glycosyltransferases in cancer malignancy is still not well defined.

In this report, we have directly shown that the glycosyltransferase C2GnT1 and its product cell membrane expressed C2-O-sLe^X^ regulate E-selectin binding and invasion of colon and hepatic carcinoma cells. After gene silencing of C2GnT1 and treatment with O- and N-glycosylation inhibitors, we observed significant reductions in the high levels of C2-O-sLe^X^ present on cells, binding to E-selectin, and cellular invasiveness into Matrigel. Binding of carcinoma cells to E-selectin was specific as evidenced by the lack of appreciable binding in the presence of a function blocking anti-E-selectin mAb. Our findings are novel because they indicate that targeting of the C2GnT1 gene and subsequent down-regulation of C2-O-sLe^X^ expression, a selectin ligand on colon and hepatic carcinoma cells, altered key properties of metastasis that are likely occur *in vivo*: decreased attachment of circulating tumor cells to endothelium and invasion into tissues. The possibility of the cellular toxicity of the gene silencing clones introduced into cells was excluded because the viability of all groups of cells was similar.

The observations reported here agree with our previous findings that C2GnT1 gene expression results in synthesis of C2-O-sLe^X^ that binds to E-selectin [25], [26]. We have found that C2-O-sLe^X^ is predominantly expressed at the advancing edge of invasive colorectal adenocarcinomas and high mRNA levels of C2GnT1 are present in these carcinomas compared to normal colonic tissues [26]. Our results support previous studies that suggest that C2GnT1 plays an important role in cancer. Earlier reports found that C2GnT1 activity is increased in human leukemia cells and metastatic murine tumor cell lines [35], [36], [37]. C2GnT1 gene expression is more highly associated with progression of colon and lung cancers than expression of sialyl Lewis structures [31], [32] and also promotes prostate cancer progression [38]. The cell surface sialyl Lewis ligands on cancer cells generated by C2GnT1 expression increase the binding efficiency of clonal cancer cell lines to E-selectin on endothelial cells which is directly proportional to their metastatic potential [39].

In this study, we did not further examine the possible mechanisms by which down-regulation of C2GnT1 and C2-O-sLe^X^ expression led to reduced invasiveness of cancer cells but we are currently investigating potential processes. We speculate that changes in C2GnT1 and C2-O-sLe^X^ expression may alter the expression and function of other adhesion molecules on cancer cells or regulate components of the extracellular matrix known to participate in invasive events. C2GnT1 may regulate the expression of tumor-associated epitopes such as cell surface associated mucins which may favor tumor progression [40]. For instance, mucins such as MUC1 and MUC4 present on cancer cells participate in signal transduction pathways that contribute to the invasive and metastatic activities of adenocarcinomas [41]. Over-expression of MUC1 is associated with invasive and metastatic colon cancer [42], [43], and MUC1 is also expressed on liver cancers [44]. Oligosaccharide and protein structures on MUC1 can mediate adhesion events by binding to E-selectin [45]. Conceivably, C2-O-sLe^X^ may be one of the oligosaccharides attached to MUC1 or another mucin present on cancer cells that may modulate their invasive properties.

Matrix metalloproteinases (MMPs) are a large group of proteins that degrade the extracellular matrix and facilitate the invasion and metastasis of malignant cells into distant tissue sites. MMPs promote motility, migration, and invasion of both colon and hepatocellular carcinomas [46], [47]. The membrane type MMPs, and in particular MT1-MMP bound to tumor cells promote cancer cell motility and invasion. MT1-MMP has also been reported to cleave MUC1 [48], and the cell adhesion molecule CD44: an E-selectin ligand that participates in adhesion and invasion [49]. MT1-MMP also participates in processing the αv subunit of integrins, which are heterodimeric transmembrane glycoprotein receptors that function as cell anchoring and signaling molecules and are also known to be involved in invasion and tumor progression [50], [51]. It is possible that C2GnT1 and/or C2-O-sLe^X^ expression in tumors may modulate the activities of MMPs and consequently their targets such as MUC1, CD44, or integrins and thereby indirectly regulate invasion.

Other enzymes can compete with C2GnT1 for the core 1 substrate and prevent the formation of core 2 O-glycans, including C2-O-sLe^X^ [52], and therefore it is important to determine the structure to which C2-O-sLe^X^ is attached. Differences in this structure may considerably influence the adhesion, invasion, and metastatic potential of cancer cells. Several scaffold molecules containing complex core 2 O-glycans, other O-glycan structures, N-glycans, or glycolipids are also terminally decorated with sLe^X^ [53], [54], [55]. We have shown here that in a human colon carcinoma and hepatic carcinoma cell line, sLe^X^ was primarily distributed on core 2 O-glycans forming C2-O-sLe^X^. Several reports indicate that high expression of C2GnT1 mRNA transcripts in carcinoma cells positively correlates with metastasis [31], [32], [33]. This report suggests that C2GnT1 activity, resulting in the generation of C2-O-sLe^X^ carbohydrates on tumor cells, in addition participates in cancer invasion. Thus, C2-O-sLe^X^ may have distinct functions depending on its site of expression: on circulating tumor cells it could enhance attachment of tumor cells to endothelium and C2-O-sLe^X^ could facilitate invasion on malignant cells that have extravasated and invaded into distant metastatic sites from the bloodstream.

Abnormal glycosylation is a prominent feature of malignant transformation and cancer progression [56]. Although our studies indicate that abundant C2-O-sLe^X^ structures present on colon and hepatic carcinoma cells mediate invasion, this moiety is not the only participant in E-selectin binding and invasion because knock-down of the C2GnT1 gene and treatment with O- and N-glycan inhibitors did not abrogate selectin binding and invasion, nevertheless, C2-O-sLe^X^ may act in concert with other molecules to promote these processes.

C2GnT1, also known as C2GnT-L (leukocyte type), is a key branching enzyme that controls mammalian core 2 O-glycan synthesis [57], [58]. Huang et al. recently reported that gene expression of another C2GnT isotype, C2GnT-M (mucin type), is downregulated in colorectal carcinomas and that C2GnT-M expression suppressed cell growth, adhesion, motility, invasion, and colony formation ability [59]. C2GnT1 and C2GnT-M may have alternate functions in regulating the progression of carcinomas at various stages of malignancy of the tumor. C2GnT-M may act predominantly as an inhibitor of early stages of colorectal carcinoma growth. As the tumor progresses to more advanced stages, C2GnT1 activity may dramatically increase resulting in a corresponding increase in C2-O-sLe^X^ expression, and increased invasion of cancer cells.

C2-O-sLe^X^ is specifically recognized by the CHO-131 mAb that has been extensively characterized in cancer and immune cells [25], [26], [28], [60], [61]. Other antibodies such as CSLEX1 mAb recognize the sLe^X^ epitope alone that can be present on several types of glycan structures including core 2 O-glycans and thus, these antibodies have broader recognition of glycan structures [62]. The reactivity of CHO-131 mAb is unique in that it requires the functional activity of the glycosyltransferases C2GnT1, α2,3-sialyltransferase, and α1,3-fucosyltransferase [28], [29], [30]. CHO-131 mAb is not a function blocking antibody, however, and at high concentrations does not inhibit interactions of C2-O-sLe^X^ terminally displayed on leukocyte PSGL1 with P-selectin [28].

In summary, our results indicate that the C2GnT1 enzyme, that regulates C2-O-sLe^X^ synthesis of cancer cells, participates in invasion of colon and hepatic carcinomas. Our findings are novel because we have identified a previously un-described function of the carbohydrate enzyme C2GnT1 and its associated product C2-O-sLe^X^ in mediating the invasive properties of colon and hepatic carcinoma cells. It is well established that inflammatory responses contribute to tumor development including the invasive behavior of tumor cells. Invasion requires extensive proteolysis of the extracellular matrix at the invasive front. Our study highlights the complex interactive roles that molecules in the tumor microenvironment play in neoplastic development. C2GnT1 and C2-O-sLe^X^ appear to contribute to tumor invasion but are not likely to be the only candidates responsible for invasive events. On carcinoma cells, C2-O-sLe^X^ may act in concert with other adhesion molecules or proteases to promote invasion and ultimately metastasis. Our results inspire additional questions about the specific mechanisms involving C2GnT1 and C2-O-sLe^X^ that mediate invasion and we are currently designing studies to directly investigate these events. We expect that our findings will impact and support the design of therapies aimed at preventing the formation of specific carbohydrates or interrupting their tumor-promoting effects in colon and hepatic cancer.

## Materials and Methods

### Cell culture

The human cell lines LS174T (colorectal adenocarcinoma) and HepG2 (hepatocellular liver carcinoma) were obtained from American Type Culture Collection (Manassas, VA) and authentication testing, including confirmation of a negative status for mycoplasma, bacteria, fungi contamination, confirmation of species identity, and cytogenetic analysis, was performed prior to purchase. Cell lines were passaged for less than 6 months after thaw for use in these studies. LS174T and HepG2 cells were grown in Minimal Essential Medium (MEM) supplemented with 1.5 g/L sodium bicarbonate, 4.5 g/L glucose, 10 mM HEPES, 1.0 mM sodium pyruvate, 1mM non-essential amino acids, 10% fetal bovine serum, and a combination of 100 U/ml penicillin/100 µg/ml streptomycin.

### Flow cytometric analysis

A FACSCanto instrument and BD FACSDiva software (Becton Dickinson Biosciences, San Jose, CA) were used for flow cytometric analyses as previously described [25], [63]. Labeling of cells (5×10^6^ cells per sample) was performed with CHO-131 mouse anti-human IgM monoclonal antibody (mAb) that detects C2-O-sLe^X^ (10 µg/ml) and was generously provided by Dr. Bruce Walcheck, University of Minnesota, St. Paul, MN or with mouse anti-human CSLEX1 IgM mAb (BD Biosciences (San Jose, CA). For the flow cytometric adhesion assays, cells were tested for binding to 10 µg/ml of a mouse E-selectin/human Fc chimera (R & D Systems, Minneapolis, MN) in buffer containing 2mM CaCl_2_ because binding is calcium dependent, and with and without 2 mM EDTA (a calcium ion chelator), or with 10 mM EDTA alone as controls. Bound antibodies were detected with a phycoerythrin (PE) conjugated F(ab')_2_ goat anti-mouse IgM secondary antibody and bound chimera was detected with a PE-conjugated F(ab')_2_ goat anti-human-IgG secondary antibody (Jackson Immunoresearch Laboratories, West Grove, PA). To evaluate the levels of background staining, cells were labeled with only the secondary antibody.

### Hydrodynamic shear flow assays

Cancer cell adhesion mechanisms were examined under hydrodynamic shear stress with a parallel plate flow chamber system (Glycotech, Gaitherburg, MD) as previously described [63]. A functional site density was defined as the minimum concentration of chimeric molecules in the correct orientation for binding required for attachment of ∼90% cancer cells at a hydrodynamic shear stress of 0.5 dynes/cm^2^ and was determined to be 0.5 µg/ml for an E-selectin/Fc chimera. LS174T colon carcinoma cells were perfused over 1 µg/ml of the E-selectin/Fc chimera under shear stresses ranging from 0.5 to 1.5 dynes/cm^2^ that were within the range previously described to occur in post-capillary venules [64]. Each assay was performed in triplicate in the presence of 10 µg/ml of an E-selectin function blocking mAb (R & D Systems, Minneapolis, MN) or the same concentration of an isotype-matched control mAb (Invitrogen, Carlsbad, CA, USA). Attached cells were enumerated at 100X magnification after 3 minutes of perfusion.

### Quantitative Reverse-Transcriptase-PCR (RT-PCR) analysis

Total RNA was extracted from 2×10^6^ cells of each cell line using Trizol reagent (Invitrogen) and DNA was removed using DNase I treatment. Reverse transcription was performed to synthesize cDNA using a First-Strand Synthesis System (Invitrogen) and 20 pmols each of primers specific for human C2GnT1, human FucT-III, and human β-actin. Quantitative real-time PCR was performed on an icycler thermocycler (Bio-Rad Laboratories, Hercules, CA) using Taq DNA polymerase (Continental Lab Products, San Diego, CA, USA) according to the manufacturer's instructions. Reactions were performed for 30 cycles with the following parameters: 94°C, 50 sec; 58°C, 50 sec; 72°C, 50 sec.

### Gene silencing of C2GnT1

A pGIPZ-C2GnT1-short hairpin (sh)RNAmir vector engineered to silence human C2GnT1 gene expression, a control pGIPZ vector expressing scrambled shRNA, four lentiviral C2GnT1-shRNA vectors in a pLK01 vector system, and an empty pLK01 vector were purchased from Open Biosystems (Huntsville, AL). All vectors were tested for silencing of the C2GnT1 gene in human hepatic carcinoma cells (HepG2) and human colon carcinoma cells (LS174T) and the vector that was most efficient at gene silencing was used in subsequent assays with each cell line. Thus, HepG2 cells were transiently transfected with a C2GnT1 shRNA-pLKO1 vector or with the empty pLK01 vector. LS174T cells were stably transduced with the pGIPZ-C2GnT1-shRNAmir vector or with the scrambled pGIPZ-shRNA vector. C2GnT1 gene silencing was confirmed in transfected or transduced cells by RT-PCR, Western blot, and flow cytometry to detect cell surface expression of C2-O-sLe^X^. The specificity of gene silencing was assessed by the use of more than one shRNA vector, by comparing the C2GnT1 and scrambled shRNA sequences to the complete human genome using NCBI Nucleotide BLAST, and by investigation of the effects of the C2GnT1 shRNA vectors on the human FucT-III gene.

### Immunoprecipitations and Western blot analyses

Whole cell lysates of LS174T cells were prepared with lysis buffer (SolObuffer-200, FabGennix International Inc, Frisco, TX) according the manufacturer's instructions. Protein concentration was quantified by the Bradford assay (Bio-Rad, Hercules, CA). Approximately 500 µg of protein was immunoprecipitated with 4 µl of a rabbit polyclonal anti-C2GnT1 primary antibody in immunoprecipitation buffer (50mM Tris-HCl, pH 8.0, 150mM NaCl, 1% Igepal, and protease inhibitor). Protein complexes were precipitated with 40 µl of Protein G beads. Products were subjected to 10% SDS-PAGE and transferred to an Immobilon-P transfer membrane (Millipore Corporation, Medford, MA), blocked with 5% blotting grade blocker non-fat dry milk, and probed with the C2GnT1 antibody or with a rabbit IgG negative control antibody and subsequently incubated with a HRP-conjugated goat anti-rabbit-IgG antibody. Protein bands were visualized with an ECL system (Pierce, Rockford, IL).

### C2GnT1 glycosyltransferase assay

The activity of C2GnT1 glycosyltransferase in LS174T cells was evaluated as previously described by Prorok-Hamon et al [65]. Cells were lysed in buffer containing 120mM NaCL, 40mM Tris, 1% Triton X-100 and 20% glycerol (Fisher, Pittsburgh, PA), and 1% Protease inhibitor (Roche Diagnostics Corporation, Indianapolis, IN). The substrate was 10 mM Gal β1,3 GalNAcα-*O*-paranitrophenyl (Toronto Research Chemicals, Downsview ON, Canada) and 10 mg/ml of proteins were used as the source of transferases.

### Treatment of cells with O-glycan and N-glycan inhibitors

HepG2 and LS174T cells (3×10^5^ cells/well) were treated with either 2mM benzyl 2-acetamido-2-deoxy-alpha-D-galactopyranoside (BGN, an O-glycosylation inhibitor), 10 ug/ml of swainosine (SWN, an N-glycosylation inhibitor), or a combination of both agents for 3–5 days. Using untreated and treated cells, the presence of membrane-associated sLe^X^ was detected with CSLEX1 mAb and membrane-associated C2-O-sLe^X^ was detected with CHO-131 mAb. For both cell types, binding to an E-selectin/Fc chimera was assessed by flow cytometry.

### Matrigel invasion assays

Twenty-four well BioCoat™ Matrigel™ Invasion Chambers (Becton-Dickinson, Bedford, MA, USA) were used for the tumor cell invasion assays according to the manufacturer's instructions. Cell suspensions (2.5×10^4^ cells/chamber) in 0.5 ml serum-free medium were added to the upper chamber of each well and 0.5 ml complete medium containing 10% fetal bovine serum was added to each bottom chamber. Chambers were incubated for 24 hours in a humidified tissue culture incubator at 37°C and 5% CO_2_. The invading cells were stained and counted in five separate fields of view at 100X magnification. Digital images were captured using a Zeiss Axiovert 200 inverted microscope and an AxioCam MRc camera using AxioVision 4.1 software (Carl Zeiss Inc., Germany).

### Statistical Analysis

Differences between groups were analyzed using a conventional two-tailed distribution Student's *t* test, with two-sample unequal variance where appropriate. Reported p-values were considered significant at p≤0.05.
